# Increased incidence of gonorrhoea and chlamydia in Greenland 1990–2012

**DOI:** 10.1080/22423982.2017.1324748

**Published:** 2017-06-01

**Authors:** Mila Broby Johansen, Anders Koch, Jan Wohlfahrt, Mads Kamper-Jørgensen, Steen Hoffmann, Bolette Soborg

**Affiliations:** ^a^ Department of Epidemiology Research, Statens Serum Institut, Copenhagen S, Denmark; ^b^ University of Copenhagen, Department of Public Health, Copenhagen K, Denmark; ^c^ Department of Microbiology and Infection Control, Statens Serum Institut, Copenhagen S, Denmark

**Keywords:** Campaign interventions, diagnostic procedures, epidemiology, sexually transmitted infections, surveillance

## Abstract

**Background**: Since the 1970s, Greenland has presented the highest reported incidence rates of the sexually transmitted infections (STIs) gonorrhoea and chlamydia in the Arctic regions.

**Objective**: This study aims to describe sex- and age-specific incidence rates of gonorrhoea and chlamydia from 1990 to 2012 in Greenland, and to evaluate if changes in case definitions, diagnostic procedures and implementation of STI interventions during the period coincide with rate changes.

**Design**: Gonorrhoea and chlamydia cases were identified from the national STI surveillance. For 1990–2008, STI cases were identified from weekly notified aggregated data. For 2009–2012, cases were identified in person-identifiable national registers. We used log-linear Poisson regression to calculate incidence rates (IRs) and incidence rate ratios (IRRs) with 95% confidence intervals (95% CI). Analyses were stratified according to sex, age and calendar period.

**Results**: Gonorrhoea and chlamydia incidence rates have increased since 1995 to reach 2,555 per 100,000 person-years (PY) for gonorrhoea and 6,403 per 100,000 PY for chlamydia in 2012. From 2006 to 2012, the incidence rates among young adults aged 15–19 years were 8,187 and 22,515 per 100,000 PY for gonorrhoea and chlamydia, respectively. Changes in surveillance reporting did not seem to influence the incidence rates for either disease, whereas a change in diagnostic test coincided with an increased incidence of chlamydia.

**Conclusion**: Overall, the incidence of chlamydia in Greenland increased during the study period, whereas the incidence of gonorrhoea decreased until 1995 but increased thereafter. Young adults aged 15–24 years were at highest risk of infection. The increase in incidence rates was independent of changes in case definitions, whereas an observed increase in chlamydia incidence in 2005 coincided with a change in diagnostic test. None of the STI interventions launched after 1995 seemed to coincide with decreasing national incidence rates.

## Introduction

Although mortality from infectious diseases has declined in Greenland since the 1950s, morbidity from infectious diseases remains a prominent public health issue, with sexually transmitted infections (STI) being of particular concern. Since the 1970s, Greenland has presented the highest incidence rates of STI among Arctic populations [1,2]. In 1975, the rate of gonorrhoea was 42,000 per 100,000 persons [2]. To reduce the high incidence rates of gonorrhoea, the Greenlandic health authorities launched focused efforts [3]. This resulted in marked reductions in incidence rates, but the decrease in rates of gonorrhoea did not continue after the 1990s. Gesink et al. showed that the incidence rates of gonorrhoea and chlamydia increased in the North American Arctic area population from 2003 to 2006, with the highest rates observed in Greenland [4].

**Figure 1.**
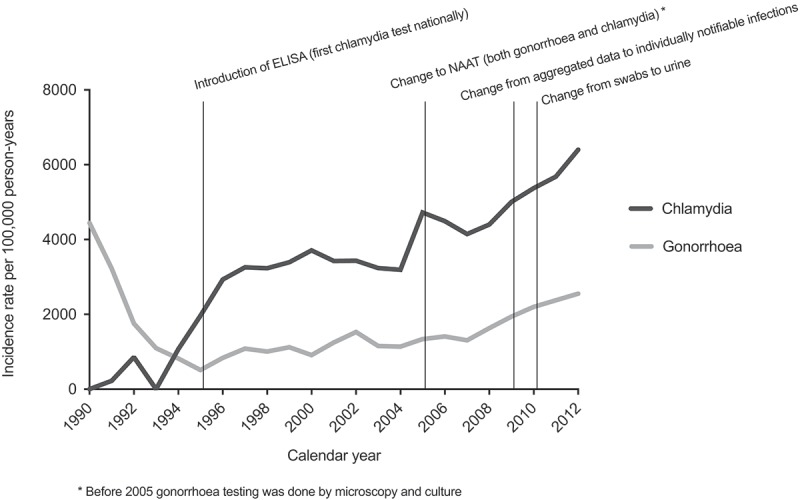
Incidence rates of gonorrhoea and chlamydia in Greenland 1990–2012 with concurrent diagnostic methods and marked year of change (2009) in surveillance system from weekly reported aggregated number to individually notifiable infections.

During the 1990s the Greenlandic health authorities have initiated several new prevention and control measures in order to reduce STI incidence rates. Introduction of new diagnostic methods, enhancement of the existing STI surveillance system and the launch of interventions such as information and screening campaigns represent such initiatives set up according to the World Health Organization’s (WHO) recommendations [5]. However, no formal evaluation of the impact of these initiatives has been conducted.

We carried out a population-based study to describe sex- and age-specific incidence rates of gonorrhoea and chlamydia from 1990 to 2012 in Greenland. Furthermore, we wanted to evaluate whether changes in surveillance methods, notification methods, diagnostic methods, and implementation of STI intervention campaigns coincided with changes in STI incidence rates over the period.

## Material and methods

### Setting

Greenland is an integral part of the Kingdom of Denmark, but with self-rule. In 2015, Greenland had a population of approximately 56,000 people [6]. The majority (89%) of the Greenlandic population are Inuit and the remaining part mostly Danes. Living conditions are comparable with those of Inuit in Canada and Alaskan Natives. Greenland provides universal and free access to healthcare to all, including medicines. Health care is provided by a local hospital in each of the 16 towns and 1 central hospital in the capital Nuuk.

### Data sources

In Greenland, every citizen is given a unique person identification number through the Civil Registration System (CRS) that follows the person from birth to death and uniquely identifies the person in national registers. The CRS register provides information on sex, date and place of birth, and continuously updated place of residence.

#### STI notifications

In Greenland, cases of gonorrhoea, syphilis, chlamydia and HIV are mandatory notifiable to the Chief Medical Officer.

From 1990 to 2008, the cumulative numbers of cases of gonorrhoea and chlamydia stratified by age and sex were reported on a weekly basis from each health district to the Chief Medical Officer of Greenland. Case definitions for gonorrhoea and chlamydia were based on clinical criteria or a combination of clinical criteria and laboratory confirmation.

In 2009, the surveillance system changed to record person-identifiable cases of STIs. Case definitions changed for both diseases to being purely laboratory based on identification of the bacterial pathogens.

#### STI sampling

In Greenland, STI testing is performed in the following situations:

(a) In case of symptoms, (b) prior to abortion, (c) prior to prescribing contraceptives, (d) in case of STI partner notifications, (e) if requested by the patient because of risk behaviour.

Pregnant women are routinely screened for syphilis, hepatitis B, HIV, *Chlamydia trachomatis* and *Neisseria gonorrhoeae* [7]. When admitted for surgical procedures, patients are routinely tested for HIV infection.

#### Diagnostic procedures

A diagnostic test for *C. trachomatis* first became available in 1994 in Nuuk and in the rest of Greenland in 1995. Until 2005, sample collection was based on cervical and urethral swabs sent to the central microbiological laboratory in Nuuk, where an ELISA (enzyme-linked immunosorbent assay) test was performed. In 2005, a NAAT (nucleic acid amplification test) based on a strand displacement amplification assay (SDA) replaced the ELISA test.

Until 2005, laboratory diagnosis of *N. gonorrhoeae* was based on cervical and urethral swabs. The gold standard method was microscopy after methylene blue staining carried out at the place of sample collection. If a positive microscopy result was obtained, culture was done at the central laboratory in Nuuk. As for chlamydia, in 2005 the diagnostic test changed to NAAT carried out at the central laboratory in Nuuk.

In 2010 sampling changed from swabbing to urine sampling for both pathogens at the majority of health clinics in Greenland. Rectal or throat swabbing is very seldom done (H.C.F. Sørensen, Personal communication, Chief district officer of Tasiilaq, East Greenland, 2016).

#### Nationwide interventions

Interventions launched during the study period were identified from the annual reports of the Greenlandic Department of Health [8], publications from the National Board of Health and Promotion (PAARISA) [9] and from peer-reviewed articles [10]. Interventions prior to 1992 were identified through peer-reviewed articles, reports and personal communication.

Interventions carried out in Greenland during 1990–2012 were categorised into groups of targeted interventions such as mass screening and case findings, and primary prevention focusing on behavioural changes. The Appendix outlines the intervention classification.

### Statistical analysis

Age-, sex- and period-specific incidence rates of gonorrhoea and chlamydia during 1990–2012 were estimated as the sum of cases divided by the sum of person-years (PY) at risk in strata of age, sex and period. For 1990–2008 the number of cases was obtained from the weekly reported aggregated lists, whereas for 2009–2012 the number of cases was obtained from the individual registrations in the STI surveillance system. Duplicate positive samples from the same person on the same day were excluded. From 1990 to 2008 PY were approximated by population size from Statistics Greenland [11]. From 2009 and onwards PY were calculated from time of residence in Greenland since 1 January 2009, until 31 December 2012, death or emigration, whichever came first. Incidence rate ratios (IRRs) and 95% confidence intervals were estimated by log-linear Poisson regression of age, sex and period-specific number of cases with logarithm of PY at risk as offset. Differences in incidence rates according to sex and age were evaluated for 3 periods (1990–1995, 1996–2005, 2006–2012) using the middle period as reference. Data was divided in the 3 periods according to changes in diagnostic procedures. The middle period was chosen as reference, as the later period is the most relevant for present interventions and the prior period represents features not relevant when comparing with the latest period. The IRRs according to period by sex and age were estimated by introducing interaction terms in the regression.

To compensate for overdispersion due to dependent events within (a person may present repeatedly with STI) and between persons (STI in one person affects the risk in other persons), variance was estimated by moment estimator with a dispersion factor based on Pearson statistics [12]. P-values were based on likelihood ratio tests. Analyses were performed using the GENMOD procedure in SAS 9.4.

### Ethical considerations

The study complies with the Helsinki Declaration II. The Commission for Scientific Research in Greenland (approval No. 2013-X086740) and the Danish Data Protection Agency approved the study (Journal No. 2008-54-0472).

## Results

### Incidence rates 1990–2012

For gonorrhoea, the incidence rate declined by 89% from 1990 to 1995 with a rate of 4,444 per 100,000 PY in 1990 to 511 per 100,000 PY in 1995 (Figure 1). From 1995 and onwards, incidence rates increased steadily to reach 2,555 per 100,000 PY in 2012.

For chlamydia the incidence rate increased during the whole study period. The most marked increase in incidence was observed from 1994 to 1997, when the incidence increased from 1,069 per 100,000 PY in 1994 to 3,257 per 100,000 PY in 1997 (Figure 1). From 1996 to 2004 the incidence was stable, while from 2004, yet another increase was observed and the incidence reached 6,403 per 100,000 PY in 2012.

As shown in Figure 1, the incidence rate of chlamydia increased in 1995, coinciding with the implementation of a national diagnostic test procedure for chlamydia, and increased sharply in 2005, coinciding with a change to a more sensitive diagnostic NAAT test. The change of surveillance system in 2009 did not seem to coincide with a change in incidence rates, as the rates began to increase before the change of surveillance. The change of diagnostic sample procedure from swabs to urine seemed to coincide with a continuously increased incidence.

Tables 1 and 2 show incidence rates (IR) and IRR stratified by calendar period, sex, and age.

**Table 1.** Nationwide incidence rates (IR) and incidence rate ratios (IRR) of gonorrhoea in Greenland 1990–2012.

		1990–1995			1996–2005			2006–2012		
		**IR**^a^ *per 100,000 PY*	**IRR**^b^ *(95% CI)*		**IR**^a^ *per 100,000 PY*	IRR *(95% CI)*		**IR**^a^ *per 100,000 PY*	**IRR**^c^ *(95% CI)*	
**Total**		1,979			1,138			1,918		
**Sex**^d^			p-value=0.02						p-value=<0.0001	
Women		1,777	1.38	(1.22–1.55)	1,133	1	(ref)	2,248	1.76	(1.64–1.89)
Men		2,159	1.66	(1.49–1.84)	1,143	1	(ref)	1,624	1.30	(1.21–1.39)
**Age (years)**^e^			p-value=0.002						p-value=<0.0001	
0–14		95	1.78	(0.88–3.63)	54	1	(ref)	138^f^	2.58	(1.70–3.98)
15–19		6,044	1.24	(1.05–1.46)	4,855	1	(ref)	8,187	1.68	(1.54–1.84)
20–24		7,223	1.50	(1.29–1.74)	4,807	1	(ref)	7,938	1.65	(1.50–1.81)
25–29		3,743	1.42	(1.19–1.71)	2,631	1	(ref)	4,096	1.55	(1.37–1.50)
30+		1,112	1.95	(1.67–2.28)	570	1	(ref)	627	1.10	(0.99–1.23)
**Sex andage**^g^			p-value=0.75						p-value=0.19	
Women	0–14	171	2.03	(1.16–3.56)	84	1	(ref)	262	3.10	(1.89–5.24)
	15–19	7,106	1.15	(0.98–1.34)	6,193	1	(ref)	11,106	1.79	(1.59–2.03)
	20–24	6,265	1.51	(1.28–1.79)	4,143	1	(ref)	8,273	2.00	(1.71–2.33)
	25–29	2,875	1.36	(1.10–1.69)	2,110	1	(ref)	4,011	1.90	(1.53–2.37)
	30+	837	1.76	(1.46–2.12)	477	1	(ref)	567	1.19	(0.98–1.44)
Men	0–14	22	0.94	(0.23–3.28)	23	1	(ref)	19	0.82	(0.19–2.94)
	15–19	5,067	1.41	(1.17–1.70)	3,587	1	(ref)	5,291	1.48	(1.25–1.74)
	20–24	8,091	1.49	(1.30–1.72)	5,425	1	(ref)	7,615	1.40	(1.22–1.62)
	25–29	4,510	1.46	(1.24–1.72)	3,089	1	(ref)	4,176	1.35	(1.12–1.63)
	30+	1,329	2.06	(1.80–2.37)	645	1	(ref)	677	1.05	(0.90–1.22)

IR, incidence rate; IRR, incidence rate ratio; CI, confidence interval.

^a^ Incidence rate per 100,000 person-years (PY).

^b^ p-values in the column for 1990–1995 represent tests comparing the age and sex specific IRRs for 1990–1995 versus 1996–2005, interaction between covariates and period.

^c^ p-values in the column for 2006–2012 represent tests comparing the age and sex specific IRRs for 2006–2012 versus 1996–2005, interaction between covariates and period.

^d^ Adjusted for age.

^e^ Adjusted for sex.

^f^ The percentage aged 13 and 14 years were 16 and 84%, respectively (based on individual registered cases in 2009–2012). In 1990–1995, 1996–2005 and 2006–2012, total events were 85, 81 and 127, respectively for persons aged 0–14 years.

^g^ Adjusted for sex and age interaction.

**Table 2.** Nationwide incidence rates (IR) and incidence rate ratios (IRR) of chlamydia in Greenland 1990–2012.

		1990–1995			1996–2005			2006–2012		
		**IR**^a^ *per 100,000 PY*	**IRR**^b^ *(95% CI)*		**IR**^a^ *per 100,000 PY*	IRR *(95% CI)*		**IR**^a^ *per 100,000 PY*	**IRR**^c^ *(95% CI)*	
**Total**		687			3,455			5,077		
**Sex**^d^			p-value=0.69						p-value=0.03	
Women		864	0.17	(0.15–0.19)	4,417	1	(ref)	6,378	1.28	(1.23–1.32)
Men		535	0.18	(0.16–0.20)	2,616	1	(ref)	3,920	1.36	(1.30–1.42)
**Age (years)**^e^			p-value=<0.0001						p-value=<0.0001	
0–14		38	0.16	(0.07–0.30)	240	1	(ref)	368^f^	1.53	(1.24–1.89)
15–19		2,955	0.17	(0.15–0.20)	17,056	1	(ref)	22,515	1.31	(1.25–1.38)
20–24		1,987	0.13	(0.11–0.15)	15,905	1	(ref)	21,241	1.33	(1.26–1.40)
25–29		1,348	0.18	(0.15–0.22)	7,341	1	(ref)	10,756	1.46	(1.35–1.57)
30+		353	0.27	(0.22–1.32)	1,316	1	(ref)	1,513	1.15	(1.07–1.22)
**Sex andage**^g^			p-value=0.99						p-value=<0.0001	
Women	0–14	64	0.16	(0.08–0.28)	401	1	(ref)	688	1.72	(1.35–2.19)
	15–19	4,263	0.18	(0.15–0.20)	24,231	1	(ref)	31,718	1.31	(1.23–1.39)
	20–24	2,312	0.13	(0.11–0.15)	17,966	1	(ref)	24,271	1.35	(1.25–1.45)
	25–29	1,540	0.19	(0.15–0.22)	8,323	1	(ref)	11,380	1.37	(1.23–1.52)
	30+	416	0.27	(0.22–0.32)	1,561	1	(ref)	1,478	0.95	(0.85–1.05)
Men	0–14	13	0.16	(0.03–0.47)	85	1	(ref)	60	0.71	(0.35–1.36)
	15–19	1,751	0.17	(0.14–0.21)	10,256	1	(ref)	13,386	1.31	(1.19–1.43)
	20–24	1,693	0.12	(0.10–0.15)	13,986	1	(ref)	18,317	1.31	(1.21–1.42)
	25–29	1,179	0.18	(0.15–0.22)	6,476	1	(ref)	10,173	1.57	(1.40–1.76)
	30+	302	0.27	(0.22–0.33)	1,117	1	(ref)	1,541	1.38	(1.25–1.52)

IR, incidence rate; IRR, incidence rate ratio; CI, confidence interval.

^a^ Incidence rate per 100,000 person-years (PY).

^b^ p-values in the column for 1990–1995 represent tests comparing the age and sex specific IRRs for 1990–1995 versus 1996–2005.

^c^ p-values in the column for 2006–2012 represent tests comparing the age and sex specific IRRs for 2006–2012 versus 1996–2005.

^d^ Adjusted for age.

^e^ Adjusted for sex.

^f^ The percentage aged 12, 13 and 14 years were 1.5, 7 and 90%, respectively (based on individual registered cases in 2009–2012). In 1990–1995, 1996–2005 and 2006–2012, total events were 34, 364 and 338, respectively for persons aged 0–14 years.

^g^ Adjusted for sex and age interaction.

The incidence rates of gonorrhoea varied significantly by sex and age over the period (Table 1). Men had the highest incidence rate during 1990–1995, and women during 2006–2012. Overall the incidence rate increased in all age groups from 1990–1995 to 2006–2012, except for persons aged 30 years or older. Persons aged 20–24 years had the highest incidence rate of gonorrhoea during 1990–1995, while the incidence rate was highest among the 15–19-year-olds during 2006–2012. Although based on very few cases, the incidence rate of gonorrhoea in the youngest age group of 0–14 year olds decreased significantly from 95 per 100,000 PY during 1990–1995 to 54 per 100,000 PY during 1996–2005, followed by a marked increase to 138 per 100,000 PY during 2006–2012. There was no significant interaction between sex and age during the 3 periods; hence, the variation in incidence by age was comparable for women and men between the periods.

For chlamydia the incidence rate was higher among women than men over the entire period 1990–2012 (Table 2). While incidence rates increased for both men and women throughout the period, the increase was similar for men and women from 1990–1995 to 1996–2005, but significantly higher for men than women from 1996–2005 to 2006–2012. The incidence rates increased in all age groups, with the 15–19-year-olds having the highest rates during the entire period. Again, while based only on few cases in the youngest age group (0–14 years) during 2006–2012, an increase in incidence rates was observed from 1996–2005 to 2006–2012, significantly more pronounced for girls than boys [IRR=1.72 (95% CI 1.35–2.19)]. In the same period a decrease was observed for boys [IRR=0.71 (95% CI 0.35–1.36)], although not significant. In contrast, a significant increase from 1996–2005 to 2006–2012 was observed in men aged 30 years or older [IRR=1.38 (95% CI 1.25–1.52)].

## Discussion

This study presents an age-, sex-, and period-specific overview of nationwide incidence rates of gonorrhoea and chlamydia in Greenland during 1990–2012. This is, to our knowledge, the most comprehensive study on gonorrhoea and chlamydia in Greenland based on nationwide figures, and the first to evaluate changes in incidence rates and their association with changes in surveillance activity, diagnostic methods, and initiation of campaign interventions.

Throughout the study period, the incidence rates of gonorrhoea and chlamydia in Greenland were among the highest in the world. In 2012, the figures from Greenland were 213 and 14 times higher, respectively, than the figures from Denmark (gonorrhoea [13]: 12/100,000; chlamydia [14]: 473/100,000), and comparable with national figures from Alaska [15] (gonorrhoea: 100/100,000, chlamydia: 749/100,000) and among high-risk female sex workers in India [16] (gonorrhoea: 5,170/100,000, chlamydia: 5,680/100,000).

The incidence rate of gonorrhoea was not consistent throughout the study period, but steadily increased for chlamydia. Gonorrhoea incidence decreased markedly from 1990 to 1995, but increased thereafter, most pronounced in the period 2006 to 2012. Yet, the incidence rate by the end of the observational period in 2012 was still lower than that observed in the beginning of the period in 1990. The highest incidence rates for both diseases throughout the study period were observed among young adults aged 15–24 years.

Prior to 1995, chlamydia was infrequently diagnosed due to lack of proper diagnostic methods nationally.

Yet, a number of factors may have influenced the incidence rates of the 2 infections, including sex, age, surveillance strategies, diagnostic procedures, and interventions.

### Sex

A constantly higher incidence of both infections was noted for women throughout the study period, except for 1990–1995 when the gonorrhoea incidence was higher among men. A plausible explanation is the sensitivity and specificity of the diagnostic microscopy of urethral secretion used in 1990–1995, which is known to be much higher among men with urethral symptoms than among women [17]. Microscopy of the cervix, rectum and throat has a low sensitivity and specificity [18,19]. Previous studies [3,20] have suggested that the high incidence rate among men in Greenland was a consequence of higher incidence among Danish immigrant workers, who tended to be male, single and >30 years of age. However, a high incidence rate was also observed among women in 1990–1995 compared with 1996–2005, and is therefore not only explained by the presence of Danish immigrant workers. We speculate whether the high incidence among women is an artefact due to the higher number tested. Whether an increase in the incidence among men would have been higher with the introduction of rectal/pharyngeal testing is unknown.

### Age

Throughout the entire period, both infections clearly occurred at a higher frequency among persons aged 15–24 years. This is not surprising and has been seen before in Greenland [21]. However, we were surprised that both gonorrhoea and chlamydia occurred in teenagers aged 14 and younger. Despite the fact that only few events of the 2 infections occurred in this age group, and primarily among 13–14-year-olds and not younger persons, it was worrying that the incidence in this age group increased from 1996–2005 to 2006–2012. Previous studies [22,23] report increased STI incidences to be associated with early intercourse, risk-taking behaviour such as less condom use, more lifetime partners and higher STI burden, especially among young women. During the past 20 years, the sexual debut of the majority of Greenlanders seems to have remained at age 14 years [24,25], although 1 report from 2010 showed earlier sexual debut (12–13-year-olds: 31% of girls, 14% of boys), with only around 50% having used condoms [24]. The explanation for the early debut of STIs may be multifactorial, including an early sexual debut and more frequent use of testing among young teenagers. As the incidence of STIs in young teenagers does not seem to decrease with time, particular attention to this group should be paid by the health system.

### Surveillance strategies

In line with the WHO’s strategy on the prevention of STIs [5] and the European Centre for Disease Prevention and Control (ECDC) [26], Greenland changed surveillance system in 2009 to a purely laboratory-based reporting system with individually notifiable notifications. Elsewhere, this approach has resulted in better coverage and more complete data on STIs [27]. There was an increase from 2009; however, a constant increase in incidence rates was already noted in the years preceding. Thus, the change did not seem to influence the incidence rates in Greenland. However, although a case diagnosis prior to 2009 might not always have been laboratory confirmed, laboratory testing had been widely used, therefore making it difficult to investigate the effect of the changes in 2009.

### Diagnostic procedures

An increase in the incidence of chlamydia coincided with the implementation of a diagnostic test for chlamydia in 1995, and again in 2005 after the introduction of NAAT instead of an ELISA-based test. Although chlamydia ELISA testing was introduced on a national scale in 1995, chlamydia incidence rates increased before that. However, the test was introduced already in 1994 in Nuuk (25% of the total population of Greenland). In addition to the fact that a number of research studies were carried out prior to 1995 and that screening efforts were carried out as part of the Stop-AIDS campaign in 1992 [28], this may have contributed to increased notification of chlamydia cases prior to 1995. An increase in chlamydia incidence with the introduction of a NAAT test is consistent with findings from the United Kingdom [29], where a 61% increase in chlamydia incidence after the introduction of NAAT was observed, explained by better coverage [30]. Change in diagnostic methods did not alone explain the increase, as the incidence increased steadily after 2005. In contrast, no effect of the change to NAAT testing was observed for gonorrhoea. The most likely explanation is that the difference in sensitivity is lower between conventional gonorrhoea diagnostics using microscopy and NAAT testing than between the first chlamydia test and NAAT [18]. Therefore NAAT has only coincided with a slight increase in the observed incidence of gonorrhoea. In 2010, urine sampling was introduced instead of swabbing. Urine sampling is preferred to swabbing by patients because of less discomfort. Furthermore, it is less labour intensive, thus allowing a larger number of persons to be tested, including asymptomatic persons [31], and may have contributed to the continuous increase after 2010. However, with the introduction of self-obtained urine sampling in Greenland, less partner notification takes place and fewer partners are tested and treated. This most likely leads to spread of infections.

### Interventions

Except for a decrease in gonorrhoea incidence during 1990–1994 under the Stop-AIDS campaign, the incidence rates of the 2 infections increased during the study period in spite of contemporary intervention campaigns. None of the intervention strategies since 1995 seemed to coincide with changes in incidence rates for both infections. However, the increased awareness of STI among patients could suggest a higher degree of testing and thereby contribute to the increase in incidences [32]. Still, it is unknown whether the incidence rates would have been higher without interventions. It was not possible to analyse effects of individual interventions, as many were not structurally evaluated and some were local and others national interventions.

In the 1960s [33] and 1970s [34] mass screenings with partner notifications and treatment guidelines according to antimicrobial resistance patterns proved to be effective. The decrease in gonorrhoea incidence under the Stop-AIDS campaign might be attributable to a multi-pronged nationwide approach: continuously intensified interventions from the 1980s onwards with both mass screenings, partner notifications, focus on sexual behaviour and information about STI, as well as preventive work with employment of a venereologist setting up venereal clinics and updating treatment guidelines. Since the Stop-AIDS campaign there have been no nationwide multi-pronged campaigns in Greenland. The interventions have mainly been based on behavioural change and local community interventions [10,35]. Low et al. emphasise a multi-pronged approach in order to combat STI burdens to target risk factors both on the individual, partnership and population level [32]. A multi-pronged approach is suggested to be effective in order to reach as many of the sexually active persons in different age groups, social and cultural levels as possible. Rink et al. and Homøe et al. also suggest that education may be effective in reducing STI incidences [10,35] and should be considered included in a multi-pronged approach.

### Strengths and limitations

The main strength of this study is the use of national surveillance and central laboratory data, which ensures completeness of STI cases in the population from 1990 to 2012. A limitation is that counting of cases during 1990–2008 was based on aggregated anonymised data. Thus, double registration cannot be excluded for this period. However, as registration was focused on persons rather than tests, we believe that only a limited number of double registrations occurred. During 2009–2012 all duplicate registrations of the same person with the same date and same disease were excluded, thus minimising the problem with double registrations of same episode. Possible double registrations within 3 weeks for gonorrhoea and 4 weeks for chlamydia were few, namely 1.5% and 2%, respectively. Obviously, some STI episodes may not have been diagnosed or reported. Differences in testing frequency between towns and settlements may be due to the difference in health care centres between towns and settlements (hospitals in towns and nurse stations in settlements). However, the high rates suggest an overall high attention and frequent health-seeking behaviour related to STIs in Greenland in the period. We therefore believe that underreporting does not play a major role in Greenland.

## Conclusion

Overall, the incidence of chlamydia in Greenland increased during the study period, whereas the incidence of gonorrhoea decreased until 1995 as part of a historical trend since 1970s but increased thereafter. Young adults aged 15–24 years were at highest risk of infection. Although cases were few, incidence rates among the 13–14-year-olds increased in particular. None of the STI interventions launched after 1995 seemed to coincide with decreasing national incidence rates. The introduction of a more sensitive diagnostic test method coincided with an increase in the incidence rate of chlamydia in 2005. Continued multi-pronged nationwide public health efforts are warranted in order to combat STI in Greenland.

## Appendix

**Appendix:** Nationwide STI intervention campaigns in Greenland 1990–2012. Mass screenings, sexual behaviour and case finding campaigns (light grey), primary prevention campaigns (dark grey).
